# An Analysis of Psychological Perceptions of Survivors of Sexual Assault

**DOI:** 10.7759/cureus.39618

**Published:** 2023-05-28

**Authors:** Sunil K Murmu, Atul S Keche, Mrinal Patnaik, Niranjan Sahoo

**Affiliations:** 1 Forensic Medicine, Pandit Raghunath Murmu (PRM) Medical College & Hospital, Baripada, IND; 2 Forensic Medicine and Toxicology, All India Institute of Medical Sciences, Bhopal, Bhopal, IND

**Keywords:** consent, rape, perceptions, survivor, psychology, sexual assault

## Abstract

Introduction: Sexual assault, although not limited to females, is predominantly a form of male-on-female oppression and a form of torture and trauma, both physical as well as psychological, and may have longstanding and lasting effects. It includes any sexual behavior or act which is threatening, violent, forced, coercive, or exploitative and to which a person has not given consent or was not able to give consent. The impact of victimization is utterly profound and there is a wide range of responses a person may have to sexual assault. Some last a few days, others a few weeks, but most can entrench far longer.

Methods: A detailed analysis was conducted on the data of each case using a standardized form and guided interview of 206 survivors who had experienced alleged sexual offenses and met the specified criteria, seeking examination at the Department of Forensic Medicine & Toxicology in a tertiary level teaching hospital in India over a period of two years. Designed as a cross-sectional observational qualitative study, relying on interviews with the survivors. The inclusion criteria encompassed survivors of alleged rape cases, kidnapping cases, and anal sex (“sodomy”) cases who presented to the department during the study period. Certain cases were excluded from the study, including those requiring only an “Ossification test” and cases involving prostitution.

Results: The experiences of 206 survivors were analyzed and we found that in the majority of cases, the assailants were known to the survivors. Reasons for this included proximity, familiarity, and taking advantage of trust and faith bestowed upon them by the victim. Up to 75.24% of the offenses were committed with consent, while 24.76% were committed without consent. The causes of consensual and forceful sex acts were explored, with the majority of consensual sex acts being attributed to false promises to marry and love affairs. The majority of non-consensual sexual offenses were forcefully committed with ill intent, with only a small number being influenced by alcohol or drug intoxication. The study also found that almost equal numbers of cases were reported by survivors and their parents, and that survivor statements were valuable for investigating cases, although there were instances where they differed from their initial statements.

Conclusion: Mental and psychological status varied among survivors, with responses related to the elapsed time period from the occurrence of the assault.

## Introduction

Sexual offenses are one such cruel kind of physical abuse that it is one of the best-known ancient forms of inflicting torture and trauma, both physical as well as psychological, and may have long-standing and lasting effects, mentally and up to some extent socially too. Sexual assault, although not limited to females, is predominantly a form of male-on-female oppression and is not an isolated event that happens to any individual in a random unpredictable way. Rather it is logical, though a completely intolerable, extension of a firmly entrenched misogynist worldview, either systematically subscribed to or imposed upon billions of people over thousands of years [1].

 Females, all over the world have to bear the problem of sexual violence (besides physical, as well as psychological) along with other oppressive societal evils [2]. Sexual assault can be any sexual behavior or act which is threatening, violent, forced, coercive, or exploitative and to which a person has not given consent or was not able to give consent [3].

Reporting the assault to the police is often not less than a daring act on the part of survivor and their family members as this invariably results in bringing the matter to the public eye, which is misogynistic and perceived to cause ‘social degradation’ and ‘loss of reputation of the family’ as far as the public is concerned [4]. Consequences of victimization in a variety of criminal cases have attracted more than adequate attention from scholars over the world. There is a wide range of responses a person may have to sexual assault. Some last a few days, others a few weeks, but most can entrench far longer [1]. It is, therefore, quite correct to state that the impact of victimization is utterly profound in cases of rape.

People who have experienced such assaults need to give themselves time to recover, reconcile with and accept that their feelings and emotions are likely to keep changing from one day to the next [4]. Each person responds to and comes to terms with the tragic experience at a different rate and in different ways, influenced by a range of factors, including but not limited to age, the circumstances of the sexual assault, their coping strategies, and response of those from whom they sought/seek support. Talking to someone about the experience soon after an assault may help people deal with its emotional impact. A psychological assessment can shed light on the emotional impact and help in understanding the mental trauma following a non-consensual sexual encounter [5].

In this study, special emphasis was laid on the psychological assessment of the survivor following the sexual assault which includes the study of the survivor’s behavior, feelings of guilt, depression, etc. so as to know the extent of suffering as described in Rape Trauma Syndrome, which is a post-traumatic panic-disorder following sexual assault. We hope this study can help guide all those involved in the investigation, medical examination and trial of sexual offenses in a compassionate and healthy manner assuring the dignity, pride, and honor of the survivors so that the survivor does not suffer from any adverse mental trauma post sexual assault and is empowered by a cooperative environment to aid in the administration of justice.

## Materials and methods

Place of study

The study was conducted at the Department of Forensic Medicine and Toxicology, M.K.C.G. Medical College & Hospital in Berhampur, Odisha. This institution serves as a tertiary center for southern Odisha, receiving cases from both Berhampur City and nearby villages.

Inclusion Criteria

The study included survivors of the alleged rape, survivors of alleged kidnapping cases, and survivors of alleged anal intercourse (sodomy) cases.

Exclusion criteria

Alleged survivors who came solely for the purpose of Ossification test, alleged survivors who refused to give consent for examination, and alleged survivors involved in cases related to PITA (Protection of Children from Sexual Offenses Act) or prostitution were not included in the study.

Study population

A total of 206 cases out of 433 cases were assessed for the study based on the aforementioned inclusion and exclusion criteria.

Study materials

The study utilized information obtained from the survivors and accompanying individuals, police requisition, treatment records of survivors admitted to the hospital, medical examination format, case record form, and x-ray requisition for ossification test when age estimation was required for the survivors. All cases were thoroughly examined using a predefined case record form according to the study requirements.

Statistical analysis

Statistical analysis of data was done by using SPSS software version 23 (IBM Corp., Armonk, NY) after complete compilation of master-chart in MS excel. The cases where data are unavailable have been excluded from statistical analysis in the respective tables. Because of the nature of assessment, the applicable tests included frequency distributions, which have been calculated from the available data in percentages. No averages or correlations have been made so p-values were not relevant to the study parameters.

## Results

Table 1 depicts the frequency of consensual and non-consensual sexual acts. Out of 206 interviewees, the majority had “consensual” sex, i.e., 154 (75.24%) and rest 51 (24.76%) “non-consensual.” Multiple acts of intercourse at multiple encounters have been considered a single increment only.

**Table 1:** Number of cases of consensual vs. non-consensual sex

Type of sex act	No. of cases	Percentage
Consensual	155	75.24
Non-consensual	51	24.76
Total	206	100

Table 2 describes the reasons for giving “consent” before the sexual act. Among various reasons, false promise to marry was the most frequent reason, with as many as 80 out of 206 (51.61%) followed next by “love affair” with 73 cases (47.10%). Notable is the fact that two of the 206 cases had been blackmailed into performing sexual acts. Although true consent precludes any form of deception or coercion, we mean the act was “consensual” in a manner such that even though it was against the wall, but with the “consent” of the survivor, in so far as they volunteered in the interview that it was consensual.

**Table 2:** Reasons behind consensual sex

Reasons	No. of cases	Percentage
False promise to marry	80	51.61
Love affair	73	47.10
Money extraction/ Black mailing	2	1.29
Others	0	0
Total	155	100

Table 3 depicts the possible reasons of “non-consensual” sex. In 42 (82.36%) cases out of 51, the act was done with forceful intention. Only six (11.76%) cases were assaulted while they were under the influence of alcohol and three (5.88%) were mentally unsound, which does not preclude temporary as well as permanent unsoundness of mind.

**Table 3:** Reasons for non-consensual sex

Reasons	No. of cases	Percentage
Forceful	42	82.36
Alcohol influenced/ Drug intoxicated	6	11.76
Mentally unsound	3	5.88
Gang rape	0	0
Total	51	100

Table 4 depicts the willingness of the survivors for medical examination by a male doctor. Of the 206 cases, 13 (6.31%) were removed from further assessment, reason being that they either could not give consent or were unable to understand the nature of examination to be conducted. Still, about half (50.49%) of survivors had no objection to examination by a male doctor.

**Table 4:** Willingness of the survivors to be examined by a male doctor

Willingness of the survivors	No. of cases	Percentage
Yes	104	50.49
No	89	43.20
Did not Consent	13	6.31
Total	206	100

Table 5 depicts the psychological profile and the feelings of the survivor at the time of examination, with shame being the most frequent (136 out of 193 cases, 70.46%).

**Table 5:** Psychological/mental status of the survivors at the time of examination

Psychological status at the time of examination	No. of cases (% age) out of 193
Feeling of Guilt	86 (44.56)
Feeling of Shame	136 (70.46)
Feeling of Humiliation	81 (41.96)
Feeling of Depression	88 (45.60)
Feeling of Fear/ Phobia	18 (9.33)
Emotionally Unstable (while the time of examination)	82 (42.49)
Absence of Readjustment to Normal Life	26 (13.47)
Suicidal attempts taken	02 (1.04)

Table 6 depicts the multitude views of survivors towards the accused even after the incidences which happened to them. Out of 206, majority (143 cases) sought marriage with the accused (69.42%), with a big portion of them (50.35%) wanting the accused to be punished if they failed to marry the survivor’s (72 cases), followed by 52 cases (25.24%) who only wished to marry. Forty nine (23.79%) wanted to punish the accused for their offenses. And six (2.91%) forgave the accused and stated they wanted the proceedings to be dismissed.

**Table 6:** Survivor’s view toward the accused

What do the survivors want now from the accused?	No. of cases (% age)
Marriage with the accused only	52 (25.24)
Marriage with accused, if not then Punishment	72 (34.95)
Marriage, if not then Punishment, or Monetary Compensation	15 (7.28)
Marriage & dismissal of the case	4 (1.94)
Punishment to the accused only	49 (23.79)
Punishment, with Monetary Compensation	8 (3.89)
Monetary Compensation from the accused only	0 (0)
Forgive & Dismissal of the case only	6 (2.91)
Total	206 (100)

Table 7 depicts the cases where FIR was lodged at police stations by the survivors themselves or anyone else. Almost equal shares of FIRs were by survivors and their parents, i.e., 93 (45.15%) and 91 (44.17%), respectively. However, in 22 (10.68%) cases, relatives lodged the FIR.

**Table 7:** Relationship of the person who lodged FIR with the survivor

FIR lodged by	No. of cases	Percentage (%)
Survivor	93	45.15
Parents	91	44.17
Relatives	22	10.68
Total	206	100

Table 8 depicts the concurrence of the statements, as stated by the survivor to us with that in the police requisition furnished. One hundred sixty (77.67%) cases were where statement was accepted as same by the survivor. However, in 46 (22.33%) cases the statements were different from what the survivor had claimed.

**Table 8:** Correlation of survivor’s statement with the requisition submitted by police

Correlation	No. of cases	Percentage
Concurrent	160	77.67
Different	46	22.33
Total	206	100

## Discussion

In the present study, the experiences of 206 cases of survivors of sexual offense were studied, out of 433 cases of alleged sexual offense cases presented for examination to the Department of Forensic Medicine & Toxicology, over the period of two years.

Contrary to our findings, studies in Ethiopia, Turkey, and Egypt reported that assailants were not known to the survivors in 42.9%, 61.9%, and 42.5% cases, respectively [6-8]. This is perhaps because of the inherent differences in study designs. In Pakistan, researchers found that only 7% cases were assailants known to the survivors which also contradicts our findings [9].

There are many reasons for the high probability of the culprits being someone known to the survivor. In male-on-female sexual assaults, her hesitance in taking positive action, being someone known to the family and the whereabouts of the person’s residence, taking advantage of the faith and confidence bestowed on them by the survivor, and opportunities for the assailants because of their proximity are the most frequently reported factors responsible for such offenses by known assailants. As far as the relatives as offenders are concerned, major factors like close proximity, staying in the same family, and known anticipation by the survivor play a great role. Other factors like social stigma, underreporting, and compromises at the base level cannot be ignored too, especially in this sub-type of sexual offenses [4].

During the study, we took utmost care to assess the motive behind all the sexual offenses by taking detailed histories from the survivors at the time of examination. A peculiarity we observed in our study was that 75.24% admitted to have committed the offense with their “consent,” whereas in 24.76% cases offense was committed “without consent.”

Our findings with regard to “consensual” sex acts is similar to the observation of a study in Bangladesh, where maximum cases were consensual too, as high as 73.86% [10]. Contradicting our result were the studies done by some researchers, who found that only 47% and 53.9% cases were consensual, respectively [11,12]. However other studies have found that most of the cases were non-consensual being as prevalent as, 69%, 68.8%, and 60.56% [13-15].

We could not find any available study specifying the reasons behind consensual and forceful sex acts. Nevertheless, we found, on further exploration of reasons behind the consensual sex act, that in the majority of cases it came to be a false promise to marry (51.61%) and love affair (47.10%). Whereas non-consensual sexual offenses in the majority cases (88.24%) were forcefully committed with only 11.76% cases under the influence of alcohol/drug intoxication. In none of the cases did we find a history of gang rape. The possible reasons for forceful sexual offense are the advantage of loneliness, seduction by the accused or taking revenge for the past conflict. Some studies documented 4%-6% cases of non-consensual sex offenses were by putting the survivor under the influence of alcohol/drug intoxication [11,16].

As far as facts of the cases are concerned, the number of cases where FIR was lodged at police stations by the survivors themselves or anyone else must be looked into as well. Almost equal numbers of cases were where the information willingly shared by survivors and their parents, i.e., 45.15% and 44.17%, respectively. Rest 10.68% cases were lodged by their relatives or caretakers. Similarly, the corroboration between the statements given by the survivors to that with the police requisition furnished has also found to be equally valuable for investigating the case. In 77.67% cases statement was accepted as same by the survivor. But in 22.33% cases they differed from their statement.

All the 206 survivors of sexual assault, when assessed for mental stability and state of emotions on the day of their medical examination, implied multiple psychological mental responses observed, possibly related to the elapsed time period from the occurrence of the assault. We found feelings of guilt (44.56%), shame (70.46%), humiliation (41.96%), fear/phobia (9.33%). This study also reveals the willingness of the survivors for examination by a male doctor, i.e., among 193 cases, was high, where more than half (53.89%) said “Yes.”

Few Indian authors have conducted the psychological assessment while examining the sexual assault survivors and found interesting results. In Manipur, a prevalence of 15.39% depression was noted at the time of examination, while 7.7% excited and majority 75% were emotionally stable [17]. Out of 206 cases, we found depression in 45.60% cases and emotional lability at the time of examination in 42.49%.

Our sample size had a high prevalence of feelings of absence of readjustment to normal life (13.47%) and two cases where suicidal attempts had been attempted (1.04%). A study from East Delhi found 4% cases were in state of acute stress reaction (absence of readjustment to normal life) [12]. Another study done in Burdwan, West Bengal, reported 28.5% were in depressed phase, 45.2% had previous history of psychiatric illness and 9.5% had suicidal thoughts [18].

It is also important to know the survivor’s intentions toward the offender after such oppression. Majority (34.95%) wished to marry the accused, and if not possible, then wanted punishment to be awarded. The next most popular intention was by 25.24% who wished to marry only, while 23.79% wanted to punish the accused for their offense, still fewer cases wanted a monetary compensation from the accused. Only six (2.91%) survivors stated to have forgiven the accused.

Limitations

There are several limitations associated with this study. The study population is limited to cases received by the Department of Forensic Medicine and Toxicology at M.K.C.G. Medical College & Hospital in Berhampur, Odisha. This may not be representative of the entire population or cases in other regions and affects generalizability. The study relies on information obtained from survivors, accompanying individuals, and police requisition and involves sensitive topics such as rape and sodomy. Due to the nature of these cases and potential emotional distress experienced by the participants, there may be inconsistencies, biases, or inaccuracies in the information provided, which can affect the reliability and validity of the study findings. It is essential to consider these limitations when interpreting the results and generalizing the findings of this study, as societal attitudes, legal frameworks, and medical practices related to these cases can change over time.

## Conclusions

In this study, the experiences of 206 alleged survivors of sexual offenses were examined and presented for examination over a two-year period. The study found that in the majority of cases, the assailants were known to the survivors. Reasons for this included proximity, familiarity, and taking advantage of the trust and faith bestowed upon them by the survivors. Delayed reporting and medical examination of cases were attributed to social stigma and prejudices, among other factors. The study found that 75.24% of sexual offenses were committed with consent, while 24.76% were committed without consent. The causes of consensual and forceful sex acts were explored, with the majority of consensual sex acts being attributed to false promises to marry and love affairs. The majority of non-consensual sexual offenses were forcefully committed with ill intent, with only a small number being influenced by alcohol or drug intoxication. The study also found that almost equal numbers of cases were reported by survivors and their parents, and that survivor statements were valuable for investigating cases, although there were instances where they differed from their initial statements. Mental and psychological status varied among survivors, with responses related to the elapsed time period from the occurrence of the assault.
